# Perspective review of what is needed for molecular-specific fluorescence-guided surgery

**DOI:** 10.1117/1.JBO.23.10.100601

**Published:** 2018-10-05

**Authors:** Brian W. Pogue, Eben L. Rosenthal, Samuel Achilefu, Gooitzen M. van Dam

**Affiliations:** a Dartmouth College, Thayer School of Engineering and Department of Surgery, Geisel School of Medicine, Hanover, New Hampshire, United States; b Stanford University School of Medicine, Department of Otolaryngology and Head and Neck Surgery, Stanford, California, United States; c Washington University in St. Louis, Department of Radiology, St. Louis, Missouri, United States; d University Medical Center Groningen, Department of Surgery, Nuclear Medicine and Molecular Imaging, Groningen, The Netherlands

**Keywords:** surgical, cancer, fluorescent, resection, therapy

## Abstract

Molecular image-guided surgery has the potential for translating the tools of molecular pathology to real-time guidance in surgery. As a whole, there are incredibly positive indicators of growth, including the first United States Food and Drug Administration clearance of an enzyme-biosynthetic-activated probe for surgery guidance, and a growing number of companies producing agents and imaging systems. The strengths and opportunities must be continued but are hampered by important weaknesses and threats within the field. A key issue to solve is the inability of macroscopic imaging tools to resolve microscopic biological disease heterogeneity and the limitations in microscopic systems matching surgery workflow. A related issue is that parsing out true molecular-specific uptake from simple-enhanced permeability and retention is hard and requires extensive pathologic analysis or multiple *in vivo* tests, comparing fluorescence accumulation with standard histopathology and immunohistochemistry. A related concern in the field is the over-reliance on a finite number of chosen preclinical models, leading to early clinical translation when the probe might not be optimized for high intertumor variation or intratumor heterogeneity. The ultimate potential may require multiple probes, as are used in molecular pathology, and a combination with ultrahigh-resolution imaging and image recognition systems, which capture the data at a finer granularity than is possible by the surgeon. Alternatively, one might choose a more generalized approach by developing the tracer based on generic hallmarks of cancer to create a more “one-size-fits-all” concept, similar to metabolic aberrations as exploited in fluorodeoxyglucose - positron emission tomography (FDG-PET) (i.e., Warburg effect) or tumor acidity. Finally, methods to approach the problem of production cost minimization and regulatory approvals in a manner consistent with the potential revenue of the field will be important. In this area, some solid steps have been demonstrated in the use of fluorescent labeling commercial antibodies and separately in microdosing studies with small molecules.

Molecular image-guided surgery has emerged as a very active research field with transformative potential to allow surgeons to see the molecular phenotype or even genotype of diseased and normal tissues during surgery.^1^^–^^5^ Much of this field’s clinical foundational roots have been developed based upon vascular perfusion imaging with indocyanine green,^6^^–^^8^ with significant growing interest in the use of other Food and Drug Administration (USFDA)-approved agents, such as sodium fluorescein, methylene blue, and isosulfan blue, which can have different transport and localization kinetics.^9^^–^^12^ Also related are the use of endogenous signals such as autofluorescence attributed to NADH/FAD concentrations, as well as absorbers such as hemoglobin and lipids^13^ and scattering agents, such as endogenous^14^^,^^15^ or surface-enhanced Raman scattering.^16^ There have also been decades of studies and approvals in photodynamic agents for diagnostic use in surgery, using porphyrins, phthalocynanines, and chlorins as uptake markers of tumors.^17^^–^^23^ A major milestone in this field just occurred, which was the USFDA approval of aminolevulinic acid-induced protoporphyrin IX to guide neurosurgery.^24^ Although already used in Europe, this action represents the first biosynthetically activated molecular probe approved for human use to guide surgery in the United States (US), named Gleolan (NX Dev Corp., recently acquired by photonamic GmbH & Co. KG). While 5-aminolevulinic acid hydrochloride (ALA) has been used in human research and approved for human use for diagnostics for decades, this recent approval comes from the result of a Phase III trial, is industry supported, and is independent of the imaging system used. This combination of features marks a very important turning point in the field. However, as a field of research in the US, surgical guidance is highly delocalized and broken down into subspecialties, without significant intercommunication, even though there is a high level of activity in each discipline. So, while many research groups are poised to achieve major discoveries and seminal studies in surgical trials, yet there is also some sense that there are major barriers to seeing this happen. This review focuses on the key aspects that require care in the clinical translation pipeline, or aspects that may be limiting the field today, and some major threats that could potentially stop the advancement of the field if not solved.

Several key factors influencing the field are shown in Table 1, broken down by analysis of strengths, weaknesses, opportunities, and threats (SWOT paradigm). Many of the strengths of the field have historically been described as needs or opportunities, but at this time, there have been major improvements that can now be counted as strengths of the field. Each of the four areas is discussed in detail in the following paragraphs.

**Table 1:** A SWOT analysis of the field for clinical molecular guided surgery.

Strengths	Weaknesses
• Robust commercial production of fluorescence guidance surgical/laparoscopy systems	• Mismatch between imaging tools today and ability to see/use biochemical heterogeneity
• Phase 0/1 trials occurring	• Over-reliance on preclinical tumors that are specifically chosen as highly positive
• First FDA approval for a biosynthetic-activated molecular surgical probe has occurred in 2017 and is being widely adopted	• Variability in clinical trial data reporting and target validation
	• Lack of standardization in analyses
	• Regulatory processes not optimally designed to assess low-dose/near-microdose agents for molecular imaging or multiple probes at one time
Opportunities	Threats
• Well-developed molecular pathology tools to phenotype biopsy tissue prior to surgery	• Misinterpretation of *in vivo* data, confusing uptake for molecular-specific uptake and how it is reported
• Proven molecular probes (metabolism, immunology, and mRNA)	• Production and toxicity tests require lower cost approaches
• Potential to save surgical time or make resection better match presurgical images	• Surgical trials inherently difficult to run due to variations between surgeons, institutional norms, and pathology processing
• Recognize close or positive margins in real-time during surgery	
• New strategies for time consuming procedures such as sentinel node mapping	• Reimbursement for intraoperative imaging not established

Arguably, one of the most important strengths of the field today is the commercial interest in providing imaging systems, leading to prototypes with strong ergonomic features for clinical use. The lack of choice and features in these systems has been a problem in the past, but today there are many systems that have been steadily improving. This success has largely been due to resurgence in the use of indocyanine green (ICG) for tissue perfusion assessment, largely driven by Novadaq Tech. Inc. (now acquired by Stryker Corp.) pushing the field forward to evolve the commercial market for this type of direct visual guidance.^25^ This has enticed production of multiple systems from manufacturers with more differentiating features to the original systems and adoption into many different surgical specialty systems.^26^ There is clearly concern about variation in system capabilities and the potential confusion of systems, and this is a topic of ongoing study in the technical performance, calibration, references standards, professional consensus, and regulatory guidance.^26^^–^^30^ This ongoing evolution and discussion at a high level of technological activity should be viewed as a strength of the field. However, much of the activity is based on anticipated success in the development of companion diagnostic agents that are highly sensitive, nontoxic, and can fit into the workflow of surgery. Similar strengths are in the early entry into exploratory uses of ICG in Phase 0/1 trials in a number of centers, as well as first in human studies of new molecules (shown in Table 2).^5^

**Table 2:** Listing of fluorescence guided surgery procedures and probes listed in clinicaltrials.gov.

Type of molecular probe	Molecule/probe	Commercial name	Clinical site or purpose (registered at www.clinicaltrials.gov)
Vascular perfusion/flow	Indocyanine green	ICG, AIM ICG	Many organ/tissue sites (>400 trials)
Lymphatic flow/sentinel nodes tissue retention	Indocyanine green	ICG	Breast, parathyroid, tumors (>40 trials)
	Methylene blue	MB-102	
Autofluorescence	NADH/FAD	n/a	Many organ/tissue sites (>190 trials)
DNA intercalation	Proflavine	n/a	Squamous cell neoplasia, Barrett’s esophagus, colon polyps, dysplasia, anal dysplasia, head and neck cancer, cervix cancer, uterine cancer, oral disorders, gastric cancer (17 trials)
Molecular vibrations	Raman scattering	n/a	Liver, macula, foot ulcers, glucose (>100 trials)
Metabolism—enzyme or synthetic activity	ALA	Gliolan	Glioma, bladder (13 trials)
		Levulan	Skin precancers and cancers (>200 mostly PDT trials)
		NPC-07	Glioma (one trial)
	Hexaminolevulinate	Cysview	Bladder, cervix, colorectal cancer (four trials)
	Methyl aminolevulinate	Metvixia	Skin AKs, cancers, Bowen’s disease, acne (68 mostly PDT trials)
	Cathepsin activatable	LUM015	Sarcoma, colorectal, pancreatic esophageal, breast, prostate cancers (five trials)
	Protease activatable	AVB-620	Breast cancer (two trials)
Metabolism—carbohydrates and proteins	Fluorescent lectin	n/a	Colorectal cancer, neoplasms, polyps (one trial)
	HSP90 inhibitor	HS-196	Solid tumors (one trial)
	Chlorotoxin blocking chloride channels with Cy5.5	BLZ-100	glioma, breast, CNS, skin, sarcoma (five trials)
	7-aa peptide—IRDye800CW	KSP-910638G heptapeptide	Gastrointestinal malignancies
	c-Met targeting peptide	EMI-137	Colon cancer, esophageal cancer and high grade dysplasia, papillary thyroid cancer, lung cancer (four trials)
Immunology—receptor and cell surface protein targeting	Folate receptor targeting	OTL38	Renal cell, lung, ovarian, pituitary, pleural cancers (nine trials)
	Tumor-specific integrin receptor binding	LS301	Breast cancer (one trial)
	Anti-EGFR binding peptide	QRH-882260	Colon cancer, cholangiocarcinoma (three trials)
	Anti-EGFR affibody	ABY-029	Glioma, sarcoma, head, and neck (three trials)
	GRPR receptor binding peptide	^68^GA-BBN-IRDye800CW	Glioblastoma (two trials)
	VEGF antibody	Bevacizumab-IRDye800CW	Esophageal, breast cancer, adenomatous polyposis (nine trials)
	EGFR antibody	Cetuximab-IRDye800CW	Pancreatic cancer, brain neoplasms, glioma, head and neck squamous cell carcinoma, head and neck cancer (four trials)
	EGFR antibody	Panitumumab-IRDye800	Pancreatic cancer, brain neoplasms, glioma, head and neck squamous cell carcinoma, head and neck cancer (four trials)
	Carbonic anhydrase IX antibody	^111^In-DOTA-Girentuximab-IRDye800CW	Renal cell carcinoma (one trial)

The key major opportunity for this field lies in the widespread adoption of molecular pathology and proteomic/genomic phenotyping of diseases in all major care centers.^31^ This means that, currently, surgeons have more knowledge than ever before about what the phenotype of the tissue is that they will be operating on, and this field is growing at an explosive rate. It is very likely that within the next decade that all tumors will have full profiling from mRNA, DNA, or proteomic assay. However, until this phenotypic profiling is truly used in surgical guidance, it remains an undeveloped opportunity. Still the idea of using the most specific proteins as carriers of molecular probes to deliver them to target tissue is extremely attractive. Particularly, the cancer therapeutics world has solid progressive growth-targeted inhibitors or proteins that could utilize a fluorescent companion diagnostic agent. The concept of adopting therapeutic antibodies as the fluorophore-carrying moiety,^32^ thereby using therapeutic biologicals as imaging agents, has already been demonstrated in ongoing surgical trials.^33^^,^^34^ Further growth along this direction is very likely going to happen and will capitalize on this approach. The opposite opportunity is also possible, that molecular imaging agents delivered prior to surgery can provide unique information about the tumor susceptibility to certain targeted drugs, susceptibility to systemically delivered agents, and appropriate therapeutic agent dosing. As the agent would be delivered prior to surgical removal, the tissue would be available for molecular, genomic, and histological examination.

The other major opportunity has been the creative growth in molecular probes for surgical guidance. A listing of those registered with clinicaltrials.org for patient recruitment is shown below in Table 2, illustrating the wide diversity of probes being tested, with a range of more and more specific binding characteristics, ranging from endogenous molecules in the beginning to exogenous probes that specially bind to cell surface carbohydrates, free proteins, specific enzymes, cellular channels, or cell surface receptors. The specificity of each is known *a priori*, and the efficacy *in vivo* is being tested in these early phase trials. At this point, most of the more specific agents have not advanced beyond Phase 1 trial, but there is potential. However, some of the factors listed next will determine success or failure of these trials.

The weaknesses of the field are the issues that will limit the successes if not solved. One of the most obvious and yet least discussed aspects of the field is that molecular-specific dyes have a localization that is often microscopically heterogeneous, and so this is not easily appreciated with surgical imaging *in vivo*. Although bulk tissue removal can still aid the surgeon as guided by the fluorescence signal, there are diseases, such as brain or ovarian cancer, where microscopic pathology removal is more critical and macroscopic views of the fluorescence will be insufficient. Pathology analysis of immunohistochemistry slides routinely shows high morphological heterogeneity in tumor nodules and ducts, indicating that specific molecular probes to these targets would likely also be microscopically heterogeneous. A pathologist is trained to view this heterogeneity and make sense of it, knowing that staining patterns are usually complex, and it is this complexity that can be one of the keys to diagnosis. Yet a surgeon must perform resection at the macroscopic or perhaps mesoscopic resolution. The macroscopic view of a heterogeneous uptake blurs out the uptake and observed contrast, and so *in vivo* levels of tumor to background are routinely seen near 1.1× to 5×.^35^^,^^36^ Whereas if the view was at the cellular or near cellular level, contrast appears at much higher levels, nearer 10× to 30× is common, but this cannot be resolved when imaging at the macroscopic scale (see examples from three different molecular tracers in Fig. 1). The difficulty lies in the basic fact that most surgical procedures perform random sampling of the tumor at the microscopic level through use of frozen sections, but do not allow for thorough microscopic examination of the tissue, and the accompanying microscopic-level image extraction. The surgeon, in general, is interested in the margin and not the complete volume of the tumor, even with all its heterogeneous components. It is the microscopic margin and the interaction with the microenvironment that presents the leading challenge to the surgeon toward macroscopic imaging. So, the field is left with a situation where the technological imaging tools used will not allow for imaging of the true disease-specific morphology, which might be better used. Perhaps even worse is the fact that as this is seemingly unrealizable, most of the focus of the field is on quantifying the available modest contrast and making decisions based upon this rather poor reporter of the true molecular specificity.^40^ An interesting side aspect of this though is that this problem is an issue for nuclear medicine molecular imaging as well as fluorescence-guided surgery (FGS), as macroscopic imaging resolution simply does not allow for delineation of the microscopic phenotypic heterogeneity of cancer, whereas in standard-of-care one might challenge if this is a real problem in the surgical theatre.

**Fig. 1:**
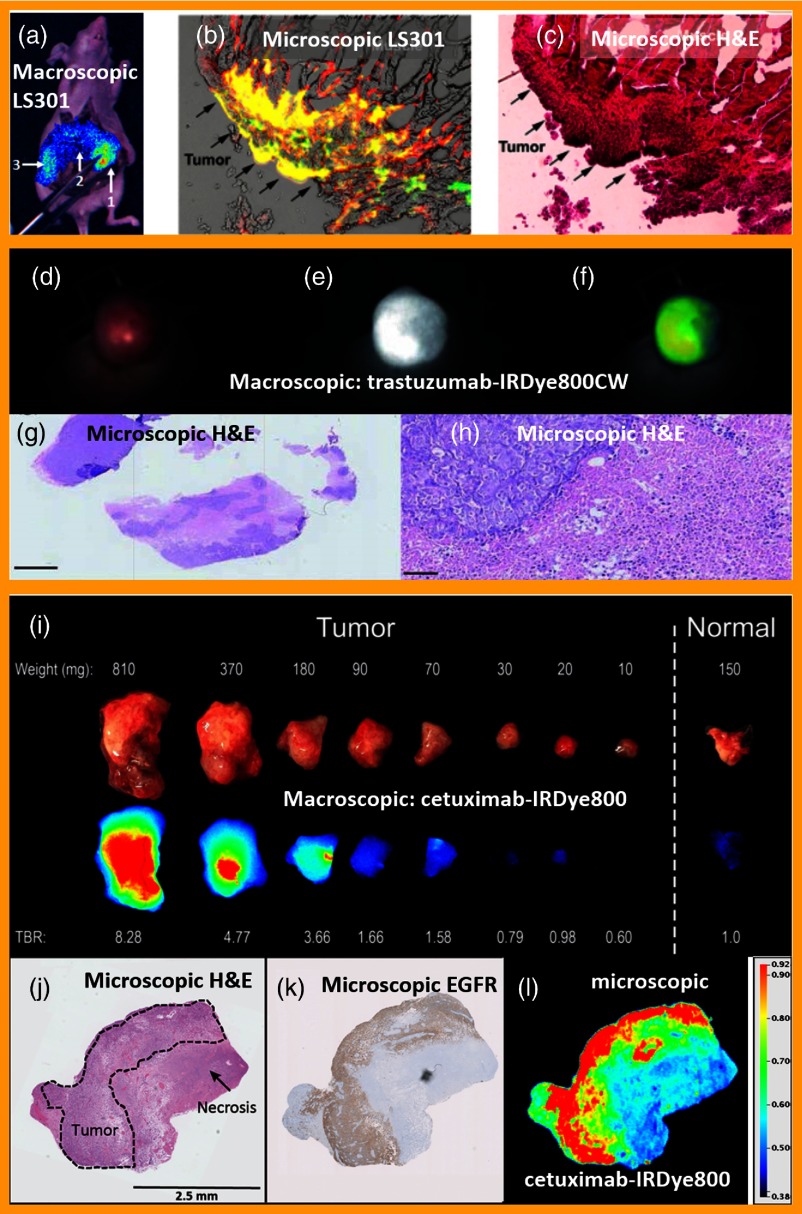
Differences in bulk contrast versus high microscopic contrast are shown. Bulk tissue imaging of NIR fluorescence image from LS301^37^ (a) overlaid on white light image of a mouse tumor, showing a fluorescence to background signal ratio, reported as 1.2 across mice. (b) High-resolution fluorescence microscopy shows colocalization (yellow) of iRFP signal (green) and LS301 fluorescence (red) exhibiting the expected high microscopic heterogeneity of the cancer. (c) Histological confirmation of the same slide showing cancerous growth corresponding to the areas marked by iRFP and LS301 fluorescence. Visualization of a tumor from trastuzumab-IRDye800CW,^38^ with a color white-light images (a), fluorescence (e) and overlay fluorescence of the two (f), with a reported tumor to background ratio of 2.7. The H&E stained tissue slides (g) and (h) show the subcutaneous tumor microscopy with heterogeneity on the 10’s of microns spatial scale, [scale bars 1 mm (g) and 50  μm (h)]. Fluorescent images of cetuximab-IRDye800CW^39^ are shown in serially cut fresh tumor (i) for different weights with a subsequent reduced TBR at each size, relative to normal tissue. The histological image and from H&E (j) and EGFR (k) are shown with the *ex vivo* fluorescent images (l) of a representative section showing the microscopic heterogeneity present in the tumor, and high labeling contrast.

One solution to imaging of the cancer heterogeneity issues, is to have a combination of: (1) a widefield high-resolution imaging system and (2) an automated software to recognize and classify the molecular morphology patterns. This would be similar to what is seen in immunohistochemistry, and automation of this is coming, but for now it remains one of the key weaknesses of the field, where the observed contrast levels seen are distinctly unimpressive. Alternatively, it is possible that fast magnification change imaging could be possible where the low power, low magnification can be quickly swept up to higher power and high resolution. The value of this is that the scoping in and out from macroscopic to microscopic imaging would allow for superior workflow for the surgeon, who desires the ability to see boundaries or fine structures better. Without these types of tools, many companies developing molecular probes may fail to see the high contrast *in vivo*, ignoring the tumor specificity it delivers in *ex vivo* analyses. As such, they might see a mismatch between *in vivo* and *in vitro* data and stop development of new molecular probes. So, while this problem can seem trivial, without a technological or logistical solution to it, there will remain a lack of clarity in target validation. What remains pivotal is comparing imaging data with the accepted gold standard in daily clinical care, which remains simple H/E histopathology. One practical issue is the comparison of frozen section fluorescence imaging to fixed H&E pathology images, which requires challenging and labor-intensive comparison studies.^35^^,^^41^^,^^42^ This is done because many targeted probes do not remain intact or active when fixed, although there are some which appear to have this characteristic. This seemingly trivial issue is a major challenge though to target verification with *in vivo*–*ex vivo* assay. Efforts to apply deep learning systems comparing *in vivo* fluorescence with *ex vivo* tumor delineation might be a solution in this complex equation. This is an important part of clinical trial testing, which without an appreciation for it could lead to a slowing of enthusiasm because of a perceived low contrast, stymying research and development in this field.

Another major weakness of the field, which pervades all of molecular cancer research, is the over-reliance of preclinical testing in tumor cell lines that are monolithically positive for the molecular target of interest.^43^^,^^44^ For example, if a single receptor directed probe is being tested, then it is tested on a tumor line that has exceptionally high expression of that receptor, because it needs to be shown that it has specificity. However, when deployed in humans, the tumor heterogeneity in terms of any protein expression can range by orders of magnitude. This preclinical approach is done for practical and economic reasons, and even though it is a widely recognized problem in cancer research, and publishers and funding agencies are trying to mitigate it, there are few obvious practical solutions.^45^ The cost of preclinical work is high, and the complexity of trying to test a molecular probe in hundreds of tumor models *in vivo* is implausible, although some methods are being proposed now.^46^ Yet, as soon as the same molecular probe is used in human trials, the range of tumors imaged can be enormously more variable than the singular xenograft models that were tested on. Additionally, the size and growth of the human tumor can alter the value of targeting, as it is well known that as tumors grow, the phenotypic characteristics can vary throughout the tumor, with variations in gene expression, protein expression, and mutated protein expression.

Given this, the ability to know the expression level and the heterogeneity of expression may be the key to allowing success in use of the molecular probe.^43^ A concern is the use of targeted fluorescence imaging in a clinical trial, often consisting of a heterogeneous patient population in terms of age, sex, tumor size, tumor heterogeneity, neoadjuvant treatment, comorbidities, etc., which might have results that are confounded by uncertain or inconclusive data about the observed target contrast, without better interpretation of both the expression and microscopic heterogeneity of this expression. Mitigating this problem could come at both ends of the pipeline, in terms of using more tumor lines in three-dimensional (3-D) culture or premade arrays, and then also better appreciating the *in vivo* pathology of each individual patient being imaged.^47^ Measurements of molecular probes though are limited to a static IHC expression, and this expression could vary over time and with sample handling. Target expression that is expressed at a low level but internalizes quickly may produce a much better signal. Furthermore, establishment of the need to identify the marker on every patient will make the process more expensive and invasive (especially, in sites like brain tumors where biopsies are rarely performed). In the end, the only current plausible solution is that the probe validation needs to be performed postsurgery by assessment of the histology/fluorescence.

Another weakness of the field is the regulatory processes that limit development and deployment of imaging probes to one at a time, with good manufacturing practices (GMP) production requirements, release testing, toxicity testing, and ongoing stability analysis for each molecule.^4^ It is possible that optimal tissue selectivity may require the type of combination targeted which is common place in clinical oncology today. This could take advantage of multiple epitopes, multiple receptors, and normal and mutated proteins, protein–carbohydrate combinations, multiple enzymes, or combinations of metabolism and immune profiles if tissue. However, the commercial barriers to imaging agents are significant,^48^^,^^49^ and even getting a single agent into approved/cleared use today seems nearly insurmountable. Thus, without a different strategy, multiple targeting moieties are unlikely to be commercially plausible. In fact, the codevelopment of a therapeutic and diagnostic agent is a conflict of interest as the returns are so lopsided; industry is often unwilling to tolerate the risk of the therapeutic agent.

The threats to FGS are perhaps the most troubling and require some careful thought before they derail progress in the field. Although each threat may not have immediate solutions, it is critical to appreciate them. The largest and most pressing threat is the fact that clinically observed contrast and uptake data are being misinterpreted at times in terms of the specificity of the signal. As previously mentioned in the weakness area, limitations of current macroscopic imaging systems can be a problem that leads to an underestimation of the specificity for the molecular probe to the target. But an even further problem that is more insidious is that macroscopic imaging tools simply cannot provide verified information about the specificity of uptake. It is very common for researchers to interpret the macroscopic contrast as the specificity of probe, and yet, factors such as vascular perfusion and lymphatic impairment can often have more effect upon the uptake than any specificity of binding or localization. This is well known as the enhanced permeability and retention (EPR) effect,^50^ and yet it is also commonly ignored when interpreting preclinical data and clinical data. This is avoided often for the simple reason that the macroscopic imaging tool cannot resolve the uptake and that the uptake patterns are mixed in with vascular perfusion patterns. Although additional target validation at the microscopic level is possible, it requires additional effort and is not always recognized by people in the development pipeline as a necessary step in the process of agent development. As a result, parsing out the vascular, localization, and lymphatic effects can be scientifically challenging and expensive to do well (Fig. 2).

**Fig. 2:**
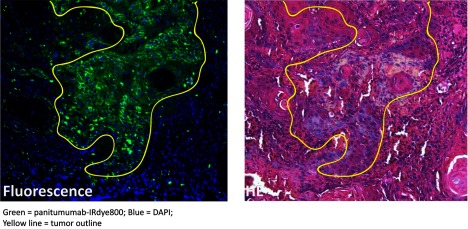
Example of microscopic target validation: panitumumab-IRDye800 localizing in head and neck squamous cell carcinoma (yellow line) but not in surrounding stromal and inflammatory tissues. This represents positive target validation but does not rule out off-target effects or failure to target all elements of cancer distributed throughout the tumor mass.

It is very common to simply compare two tumor lines, one positive for the target, and one negative for the target as a quantitative assay of specificity of uptake. Yet, while this seems reasonable, these two tumor lines can easily have much larger vascular perfusion and lymphatic flow differences, which will dwarf the effects of the probe binding. Preclinical testing of specificity in this manner, while seemingly logical, based just on the target, adds a high level of randomness to the field, and many published studies have data that are confounded by this issue. Solutions to this problem are complex and require more careful microscopic and pathologic analysis of uptake, comparing matched targeted and untargeted probes, to ensure that the measured bulk uptake is actually bound to the target or activated by the expected target. Solutions such as using a reference tissue to normalize are insufficient,^51^ because they do not solve the problem of the specificity of uptake. Modeling studies,^52^^,^^53^^,^^54^ detailed immunohistochemistry studies, or comparison with a paired tracer can each potentially mitigate the problem,^55^^,^^56^ but are more easily implemented at the preclinical stage than the clinical stage.

Another key threat is the lack of an obvious way to advance production and *in vivo* toxicity testing in a way that allows the cost to match the benefit.^48^^,^^49^ The hardest part of this as the field evolves is having examples of business cases that allow for companies to recoup their expenditures for the development cost. Current GMP (cGMP) production of small organic dyes for human use have been pioneered by a few groups, such as the work of Hyun and colleagues.^57^ Conventional contrast agents have been reducing in numbers for most of the last decade,^58^ and the development of new CT and MRI contrast agents is comparatively stagnant, and this is even true in nuclear medicine. This is related to a lack of additive potential revenue from their use. This is a challenging problem, although solutions may exist in following a few of the case examples in nuclear medicine,^2^ taking advantage of the use of cGMP methods in academic laboratories with initial human testing in microdose trials.^59^^,^^60^ Perhaps the most common method of using this research-based approach, especially when there is limited return but high value to the patient, is the development of registries supported by the Centers for Medicare and Medicaid Services. This method was critically successful in molecular imaging, providing for the development of FDG-PET imaging, and seems a viable pathway, as long as the imaging systems used are sufficiently sensitive enough to image nanomolar levels of agent.^29^^,^^60^^,^^61^^,^^62^ Other solutions may also emerge as the field develops, such as gaining approvals for mixtures of agents at a time, although this is seemingly harder to imagine as time goes on.

Toxicity and release testing will always be a necessary and expensive part of the process as well, which cannot be avoided. However, there are methods that can limit costs, if, for example, the imaging is done with therapeutic proteins that have already undergone substantial human use, and can be slightly modified without altering their pharmacokinetic behavior. In these cases, minimal testing might be feasible under negotiation with regulatory bodies.^4^ Alternatively, microdose studies are also possible,^59^^,^^60^^,^^63^ which reduce the required toxicity studies to a single rodent species with a single injection, from the more complex multiple-species-multiple-dose studies. This can be effectively completed for a modest cost and can even be completed on the test batch of an agent instead of the final product, as long as there is consistent good laboratory practice followed between the production stages. Toxicity testing will become a larger aspect of agents as they expand and while this is not a biomedical optics problem *per se*, there are approaches such as *in vivo* pharmacokinetic imaging,^64^ which might be deployed to reduce animal use and cost. Similarly, *in vivo* optical imaging to assess activity and target bioavailability will likely be useful to confirm the value of agents.^65^ Finally, better understanding of the *in vivo* kinetics and clearance organ behavior of both the dye and the targeting moiety can alleviate some of the costs of preclinical work,^66^^,^^67^ where some are developed without sufficient planning for avoiding organs at risk or appropriate clearance kinetics.

The threat about economic viability for new molecular probes is less of an issue for small molecules than it is for larger biological agents, and it is likely that the two will have very different development paths. It is possible that once the safety of fluorescently labeled therapeutic antibodies has been better established, one could make the case that small labeling changes are relatively minor, and that these agents, if made under certain parameters, should inherently have FDA clearance if the antibodies are already in common human use.^4^ This is valid if the fluorescent labeled antibody has the same biological clearance and activity as the native antibody, because with careful labeling there is minimal effect to a 155-kDa antibody from a 0.5-kDa dye added to it. This has been done by several groups in ongoing clinical trials in the US, with Cetuximab and Panitumumab,^68^^,^^69^ and in Europe, with bevacizumab and an anti-CEA antibody.^70^ The application of this approach to developing agents for multicenter clinical trials has not been achieved yet, nor to FDA cleared products, but it is a promising pathway.

## Summary

The perceived value of molecular guided surgery is exceptionally high. It comes with the promise of combining molecular pathology information into a nuclear medicine-like molecular guidance, which would improve the precision of surgical procedures. Increasing interest by different surgical specialties has been robust,^3^ matching the commercial investment occurring. To keep this field progressing to its highest potential, the issues mentioned in the weaknesses and threats areas need attention and creative input from partners in engineering, medicine, and industry.
